# “One-step” approach versus “Step-up” approach minimally invasive treatment for infected pancreatic necrosis: a study protocol for a single-center, prospective, randomized controlled trial

**DOI:** 10.1186/s12876-022-02116-0

**Published:** 2022-02-03

**Authors:** Zhi Zheng, Jiongdi Lu, Feng Cao, Yixuan Ding, Yulin Guo, Wentong Mei, Yuanxu Qu, Shuang Liu, Haichen Sun, Yeqing Cui, Ang Li, Fei Li

**Affiliations:** 1 Department of General Surgery, Xuanwu Hospital, Capital Medical University, No.45 Chang-chun street, Xi-Cheng District, Beijing, 100053 China; 2 Clinical Center for Acute Pancreatitis, Capital Medical University, Beijing, China

**Keywords:** Acute pancreatitis, Infected pancreatic necrosis, Minimally invasive treatment, Randomized controlled trial

## Abstract

**Background:**

Currently, the minimally invasive “Step-up” surgical strategy is still the main treatment for infected pancreatic necrosis (IPN). However, indiscriminate implementation of the “Step-up” strategy can lead to increased numbers of operations and prolonged hospital stay. The “Step-up” approach is not appropriate for some patients due to unavailabilty of a safe puncture path. Therefore, we developed the “One-step” surgical approach to treat IPN, which is safety. However, there is still a lack of comparison of the short and long-term efficacy between the “One-step” and “Step-up” approach. Consequently, we are conducting this clinical trial to provide a reference for IPN treatment.

**Methods:**

This is an ongoing, single-center, randomized controlled trial of patients with IPN. The total sample size required for the trial (May 2021–December 2023) is approximately 128 patients. Patients will be randomly assigned to either an experimental group (One-step) or a control group (Step-up) at a ratio of 1:1 using the block randomization method. We used the case report forms and electronic data capture systems to obtain demographic information, preoperative laboratory examination, auxiliary examination results, surgery data, postoperative recovery outcomes, and follow-up outcomes. The patients will be followed up for 2 years after surgery. The primary endpoint is a composite endpoint, consisting of mortality and severe complications. The secondary endpoints include the incidence of organ dysfunction, the number of surgical procedures, mortality (the incidence of death in hospital and deaths within 30 days of discharge), hospital stay, intensive care unit stay, hospitalization costs, perioperative inflammatory marker changes, and short-and long-term complications.

**Discussion:**

Compared with the “Step-up,” the “One-step” minimally invasive surgery can significantly reduce the number of operations, reduce the length of hospital stay and hospitalization costs without increasing the incidence of composite endpoint events, and has better short- and long-term efficacy and safety. Additionally, there was no statistically significant difference in perioperative complications and mortality between “Step-up” and “One-step”. This study will assist with the formulation of an effective and scientific “One-step” minimally invasive treatment strategy for IPN, and an understanding of this technique will facilitate clinical decision-making for IPN.

*Trial Registration* ChiCTR2100044348. Trial status: Ongoing.

## Background

Severe acute pancreatitis (SAP) is a serious abdominal disease with a poor prognosis and high mortality rate [1]. There are two mortality peaks in SAP: (1) systemic inflammatory response syndrome (SIRS) and multi-organ dysfunction (MODS) in the early stage of the disease; (2) infected pancreatic necrosis (IPN) occurs in the late stage of the disease, and its incidence in the natural course of SAP is as high as 40–70% [2]. With the advancement of fluid resuscitation and other treatments, the early mortality rate has been reduced, but the treatment of IPN is still very difficult. Approximately 35–50% of SAP deaths are related to IPN surgical intervention is often required, and the outcomes of treatment are crucial to prognosis [3]. Therefore, the treatment of SAP, especially for IPN, has always been a major issue and a research hotspot in the field of digestive diseases.

In recent years, minimally invasive pancreatic necrosis tissue debridement has become the primary treatment for IPN, and most clinicians follow the “Step-up” surgical strategy, namely percutaneous catheter drainage (PCD) is performed first, and minimally invasive or open surgery are performed successively for patients for whom infection is not controlled [4–6]. However, indiscriminate implementation of the “Step-up” strategy can lead to increased numbers of operations and prolonged hospital stay, as the “Step-up” approach is not appropriate for some patients due to unavailability of a safe puncture path, causing increased trauma to patients [7]. Therefore, we developed and currently apply the “One-step” surgical approach to treat IPN, that is, direct minimally invasive debridement instead of PCD. A preliminary retrospective study data from our center confirmed that “One-step” has better surgical efficacy and safety, but there is a lack of clinical data comparing it with the “Step-up” approach [8]. Consequently, this study is intended to conduct a single-center, randomized controlled trial to compare the “Step-up” with the “One-step” approaches in terms of the composite endpoints which consist of mortality and severe complications to provide a reference for the distribution characteristics of IPN and the selection of minimally invasive surgical procedures. In accordance with the SPIRIT reporting checklist, we present the following article.

## Methods/design

### Study design and setting

**Fig. 1:**
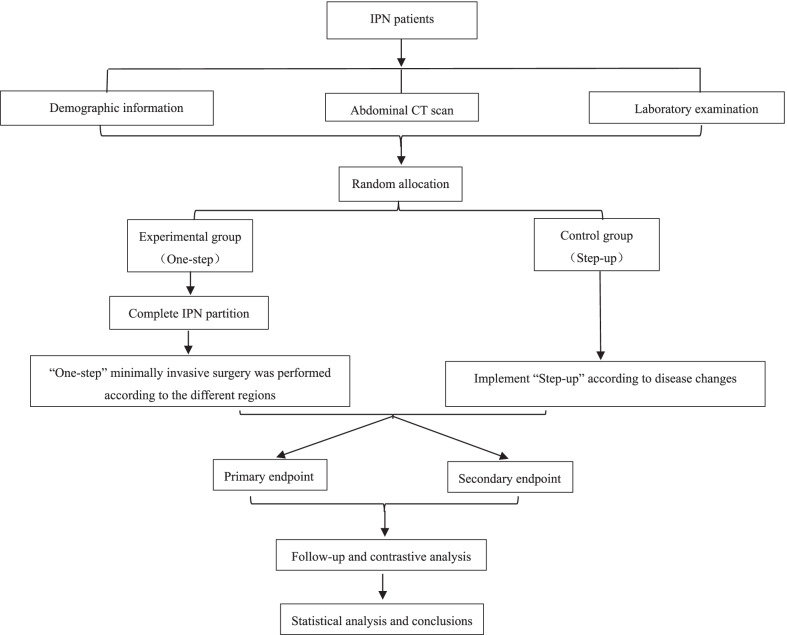
Research process flow chart

This study is a single-center, randomized controlled trial. The research began on May 1, 2021, and is anticipated to end on December 31, 2023. During the research period, patients will be selected from Xuan Wu Hospital, Capital Medical University for treatment. A total of 128 patients will be enrolled in this study. After providing informed consent, enrolled patients will be randomly assigned for surgical treatment to either the experimental group (One-step approach) or the control group (Step-up approach) at a ratio of 1:1 using the block randomization method. Each patient will receive a numeric randomization code. The detailed research process is illustrated in Fig. 1.

### Ethics approval and informed consent

This study has been approved by the Ethics Committee of Xuan Wu Hospital, Capital Medical University. The ethical approval number is 2020-052. According to the requirements of the Ethics Committee, clinical research will be conducted only after the enrolled patients have signed informed consent forms. This study was designed in accordance with the principles of the Declaration of Helsinki (as revised in 2013). All data will be recorded and analyzed anonymously to protect patient privacy. The trial was registered in March 2021 on the Chinese Clinical Trial Registry website (http://www.chictr.org.cn/index.aspx); registration number ChiCTR2100044348. Participating patients will be informed of the purpose and significance of the study, the benefits and possible risks of participation, and the confidentiality of the study.

### Inclusion criteria

1. The patient was diagnosed with acute pancreatitis with infected pancreatic necrosis by abdominal computed tomography (CT) scan and laboratory examination, (e.g. CT scan showed “bubble” sign or bacteria or fungi are detected by fine-needle aspiration and culture).
2. Infected pancreatic necrosis mainly included acute necrotic collection with infection, walled-off necrosis with infection, and isolated infected peri-pancreatic necrosis.
3. Patients are between 18 and 80 years old, regardless of sex.
4. The patient’s general condition is stable without multiple organ failure (according to the modified Marshall score).
5. The patient was not treated with PCD or the target infected area was not treated with PCD.
6. The patient was qualified for video-assisted minimally invasive debridement and signed the informed consent for treatment.
7. The patient is capable of following the study protocol and follow-up scheme.

### Exclusion criteria

1. Previous history of pancreatic necrotic tissue drainage or debridement
2. Previous exploratory laparotomy for acute abdominal disease or pancreatitis
3. The patient experienced acute exacerbation of chronic pancreatitis
4. Patients with acute pancreatitis with abdominal compartment syndrome or abdominal organ perforation
5. Patients with acute pancreatitis due to abdominal surgery
6. Patients who cannot tolerate video-assisted minimally invasive debridement and anesthesia due to physical condition
7. Patients participate in other clinical studies.

### Elimination criteria

1. The patient cannot comply with randomization.
2. The patient’s clinical data were incomplete and could not be statistically analyzed.
3. Researchers eliminated enrolled patients from study for patient’s benefit.
4. The patients violated the study protocol and received other therapies during the study period.

### Participating surgeons

We have selected three experienced pancreatic surgeons and several nurses on our medical and research team available to perform surgeries and postoperative care for enrolled patients. The clinician performing the operations will be required to have an associate senior or senior title and have completed at least 30 laparoscopic or open pancreatic necrotic tissue drainage or debridement procedures, indicating their capacity to handle emergencies during the operation and ensure the treatment quality for enrolled patients.

### Primary outcome

The primary outcome is the composite endpoint, which consists of mortality and severe complications (Clavien-Dindo ≥ IIIa). According to the Clavien-Dindo classification, postoperative complications classified as higher than Grade IIIa will be regarded as clinically significant (Table 1) [9].

**Table 1:** Clavien-Dindo Classification

Grade	Definition
I	Any complication that deviates from the natural course after surgery, including the use of antiemetics, antipyretics, analgesics, diuretics, infusion, and physical therapy, as well as bedside debridement of incision infection
II	Medications other than those permitted for grade I complications are required, including blood transfusion and total parenteral nutrition support
*III*	Surgical, endoscopic, and radiotherapy are required
IIIa	No general anesthesia is required
IIIb	Need for general anesthesia
*IV*	Life-threatening complication, including centeral nervous system complications and required intermittent monitoring or intensive care unit (ICU) treatment
IVa	Single organ dysfunction
IVb	Multi-organ dysfunction
V	Death

### Secondary outcome

1. The incidence of organ dysfunction and organ function were evaluated according to the modified Marshall score.
2. Complication: The complication rate mainly includes short-term and long-term complication rates, among which short-term complications include bleeding, gastrointestinal perforation, and pancreatic leakage. Long-term complications include incisional hernia, pancreatic endocrine and exocrine dysfunction, chronic pancreatitis, and portal hypertension. Pancreatic endocrine dysfunction was defined as HbA1c ≥ 6.5% (48 mmol/mol) and/or FPG ≥ 126 mg/dl (7.0 mmol/l) 3 months after diagnosis or pancreatitis. Pancreatic exocrine dysfunction was determined in a variety of ways including direct pancreatic function testing, measurement of fecal elastase or fecal fat levels (72 h fecal fat excretion > 7 g/d) and need for oral pancreatic enzyme replacement therapy. Chronic pancreatitis was defined as repeated upper abdominal pain; abnormal serum or urine pancreatic enzyme concentrations (lipase or amylase activity 2–3 times above the upper limit of normal); abnormal exocrine function (stool elastase < 200 µg/g) while the endosonography/CT/MRI imaging shows dilated main duct and side branches, intraductal and parenchymal calcifications[10, 11].
3. The number of surgical procedures which includes PCD and the total number of operations.
4. Mortality: The incidence of in-hospital death and deaths within 90 days of discharge.
5. The length of hospital stays, the length of intensive care unit (ICU) stay, and hospitalization costs.
6. Changes in inflammatory markers, including white blood cell (WBC), interleukin-6 (IL-6), C-reactive protein (CRP), and procalcitonin (PCT)

### Interventions

In this study, we compare the surgical treatment for IPN which include “One-step” and “Step-up” minimally invasive pancreatic necrotic tissue debridement. Although, the endoscopic transmural (transgastric or transduodenal) necrosectomy was also considered as an effective treatment for IPN [12], it was not routinely used in this study.

### “One-step” minimally invasive pancreatic necrotic tissue debridement

This method is a direct, minimally invasive surgery, omitting one of the surgical procedures of percutaneous catheter drainage (PCD). According to the preoperative imaging examination, “One-step” minimally invasive pancreatic necrotic tissue debridement can be performed via the omentum sac or retroperitoneal approach.

Since the IPN partition is directly related to the surgical approach, clinicians need to determine the type of IPN partition by abdominal CT scan prior to “One-step” minimally invasive pancreatic necrotic tissue debridement. The surgical procedures for the two different surgical approaches and IPN partition are described in Table 2.

**Table 2:** The IPN distribution and the selection of surgical approach

Region	IPN distribution	Surgical approach
I	Peripancreatic area†	Median abdominal approach
*II*		
IIa	Left posterior colon area‡	Median abdominal approach or left retroperitoneal approach was used to drain the head of the pancreas
IIb	Left pelvic area§	Left retroperitoneal approach was used to drain the caudal side of the pancreas
III	Right posterior colon area¶	Right retroperitoneal approach

^†^ Peripancreatic area includes intraomentum, pancreas, transverse colon, and mesentery root, perisplenic, and left subdiaphragmatic area

^‡^ Left posterior colon area includes left posterior colonic space, the anterior and posterior space of left pararenal, and the perirenal space, but the pelvis cavity is not involved

^§^ Left pelvic area includes the anterior and posterior space of rectum

^¶^ Right posterior colon area includes the posterior space of duodenum, posterior space of right colon, anterior and posterior space of right pararenal, and right perirenal space

#### Omentum sac surgical approach

(1) Incision selection: The preoperative CT scan will be carefully evaluated, and the boundary of the peripancreatic abscess will typically be marked by the xiphoid process or umbilicus. The incision should be selected in the midline nearest to the abscess, and the stomach and transverse colon should be avoided as far as possible. The incision length is usually approximately 5 cm. (2) Passage is established through the omental sac: After entering the abdominal cavity, the gastrocolic ligament is incised below the vascular arch of the gastroepiploic artery. Bleeding caused by injury to the gastroepiploic vessels must be thoroughly sutured to avoid postoperative bleeding in the abdominal cavity or the omental sac. The gastrocolic ligament and parietal peritoneum are sutured around the circumference to establish access and protect the abdominal cavity from pus inflow. The location of the abscess cavity will be determined by fine-needle puncture (application of intraoperative ultrasound positioning) and bluntly separate into the abscess cavity. (3) Video-assisted pancreatic necrotic tissue debridement: After the tissue is collected for bacterial culture, pus is absorbed as much as possible, and the abscess cavity is observed through video equipment. Under video guidance, the necrotic tissue is removed with an aspirator, oval forceps, or laparoscopic instrument, blood vessels are carefully protected, and immature necrotic tissues are preserved. (4) Place drainage tube: The drainage tube is placed under laparoscopy and the location and number of placements depend on the location and size of the abscess. It is recommended to use a 30–36Fr three-chamber drainage tube to reduce the risk of blockage.

#### Retroperitoneal surgical approach

(1) Incision selection: Carefully evaluation will be made of the results of the CT scan before surgery, and the midaxillary line is usually used as the body surface marker. The incision should be selected near the midaxillary line closest to the abscess (surgery can be performed through the left or right midaxillary line) while avoiding the ascending colon, duodenum, and descending colon as much as possible. The incision length is usually approximately 5 cm. (2) The retroperitoneal passage will be established: Incise the skin, subcutaneous tissue, and abdominal wall muscle, and hemostasis of the abdominal wall muscle should be thorough to avoid postoperative wound bleeding. After entering the peritoneum through the retrocolic space, the abscess cavity will be determined by fine-needle puncture and then bluntly separated into the abscess cavity. Video-assisted pancreatic necrotic tissue debridement and drainage tube placement are the same as those in the omentum sac surgical approach. Surgery can be performed through the left and right midaxillary line or bilateral approaches. The first operation is usually performed under general anesthesia with tracheal intubation or laryngeal mask, and the second operation may be performed under local anesthesia depending on the abscess and the general condition.

### “Step-up” minimally invasive pancreatic necrotic tissue debridement

“Step-up” minimally invasive pancreatic necrotic tissue debridement which uses PCD as the initial treatment option for IPN. The retroperitoneal approach is the preferred approach for PCD treatment under the guidance of B-ultrasound or abdominal CT scans. “Step-up” minimally invasive surgery should be considered if the patient’s infection symptoms do not resolve within 48 h following PCD treatment. The surgical approach is the same as the “One-step,” and the surgical approach was retroperitoneal or omentum sac, depending upon the path of the PCD drainage tube and preoperative imaging examination. If there is residual infection in the abdominal cavity after minimally invasive surgery, PCD treatment is preferred. Open surgery is only used as the last rescue measure after the failure of minimally invasive surgery.

### Perioperative treatment for enrolled patients

For patients who received “One-step” minimally invasive pancreatic necrotic tissue debridement, clinicians must provide symptom-based treatment, such as electrocardiograph monitoring, antibiotics, proton pump inhibitors, analgesics, octreotide, and nutrition support. Routine blood tests, serum CRP, PCT, IL-6, and arterial blood gas tests will be used to monitor delayed bleeding, incision, and/or abdominal infection after surgery. After the recovery of gastrointestinal function, a semi-liquid diet can be gradually administered until a normal diet is resumed. In addition, it is also necessary to pay attention to the characteristics of the drainage fluid in the abdominal drainage tube and be alert to delayed abdominal hemorrhage. When the amount of drainage fluid in the abdominal drainage tube is gradually reduced and the characteristics of the drainage fluid are clear, the drainage tube can be removed gradually. The patients can then be discharged from the hospital.

For patients who received “Step-up” minimally invasive pancreatic necrotic tissue debridement, clinicians provide electrocardiograph monitoring, antibiotics, proton pump inhibitors, analgesics, octreotide, and nutrition support after receiving PCD therapy. Routine blood tests, serum CRP, PCT, IL-6, and arterial blood gas tests are continued to monitor the abdominal infection. If the patient’s infection symptoms are resolved, the current therapies will be continued until normal status is achieved. If the patient’s infection symptoms are not relieved within 48 h of PCD treatment, they will undergo minimally invasive pancreatic necrotic tissue debridement. The postoperative treatment is the same as the “One-step” approach.

### Assignment of intervention for allocation

#### Sequence generation

To ensure the matching of clinical data between the two groups, the participants will be randomly assigned to the experimental group and the control group by block randomization. SAS 9.2 software (SAS Institute Inc., NCSU, USA) will be used to generate the randomization sequence and each patient will be assigned a numeric randomization code.

#### Allocation concealment mechanism

Patients will be randomly assigned to the experimental group (One-step approach) and the control group (Step-up approach) at a ratio of 1:1 using the block randomization method.

### Implementation

The Contract Research Organization in Xuan Wu Hospital will generate the allocation sequence, enroll participants, and assign participants to interventions.

### Assignment of interventions for blinding

The study is a single-blind trial in which the patients will be blinded to their grouping. However, the surgeon will provide the corresponding surgical treatments according to the grouping of patients. After patients are discharged from the hospital, clinicians will send the surgical methods in the form of medical records through the Email.

### Data collection

Clinical data of enrolled patients will require collection through a case report form (CRF) and the electronic data capture (EDC) system. Every month, clinical research associates (CRAs) monitor the electronic database to guarantee data quality when the clinical research coordinator (CRC) enters the clinical data into the EDC system from the CRF.

The CRF includes the following data: (1) Demographic information: sex, age, body mass index, concomitant disease, and medication use; (2) Perioperative laboratory examination: results of routine blood tests, biochemical tests, serum CRP test, PCT, IL-6, and arterial blood gas tests; (3) Auxiliary examination: all enrolled patients will undergo abdominal enhanced CT scans; (4) Surgery data: operation date, operation time, number of operations, blood loss, surgical methods, and intraoperative complications; and (5) Postoperative recovery outcomes: hospital stay, length of ICU stay, organ dysfunction, perioperative blood pressure, and heart rate, hospitalization cost, short-term and long-term postoperative complications, and death (Table 3).

**Table 3:** Checklist for the collection of necessary clinical data and follow-up scheme for enrolled patients with IPN

	Baseline		Post-operation				Follow-up					
	Pre-operation	Operation	POD 1	POD 3	POD 7	POD 30	3 months	6 months	9 months	12 months	18 months	24 months
Inclusion/exclusion criteria	×											
Informed consent	×											
Allocation	×											
Demographic information	×											
Laboratory examination	×		×	×	×	×	×	×	×	×	×	×
Auxiliary examination	×						×	×	×	×	×	×
Surgical data		×										
Postoperative recovery outcomes			×	×	×	×						
Physical examination	×						×	×	×	×	×	×

× indicates the need to collect clinical data

POD, postoperative day

The important privacy information of patients will be replaced with statistical codes or numbers, then CRC will transfer the data from CRFs to the EDC system (https://edc.trialdata.cn/Member/Login), and all clinical data will be analyzed anonymously via the EDC system. Detailed results will be openly shared with the permission of the corresponding author at the end of the study.

### Follow-up

After the procedure, a full-time staff member will be responsible for the follow-up of the enrolled patients. Each patient will be followed up for two years after discharge by outpatient or inpatient review, telephone, or mail. Patients will be followed up every three months in the first year after surgery and every six months over the second year after surgery. During the follow-up period, patients are required to undergo physical examinations, abdominal CT scans, and laboratory tests. In addition, researchers will monitor for related clinical symptoms, such as abdominal pain, bloating, and fatty diarrhea. Physical examination will be conducted mainly to check for postoperative incisional hernia. Abdominal CT scan will primarily focus on the morphological changes of the pancreas and blood vessels in the abdominal cavity. Laboratory tests will include routine blood tests and blood biochemistry tests, which are concerned with white blood cells, thrombocytes, blood glucose, blood triglycerides, and glycosylated hemoglobin. The detailed follow-up schedule is shown in Table 3.

### Adverse events

All serious adverse events (SAEs) occurring between the signing of the informed consent and the end of the trial will be recorded in detail within 24 h. Subsequently, researchers will report the SAEs to the ethics committee of the unit. SAEs are defined as any damage whether associated with the anticipative outcome of the surgery or not. The study includes a data monitoring committee that will supervise the safety data in an unblinded manner in accordance with the Standard Operation Procedures for Clinical Trials. Enrolled patients will receive the best treatment available to resolve any complications.

If the incidence of treatment-related deaths or the proportion of Grade IV postoperative complications determined to be causally related to the procedure exceeds 5% of the total number of patients enrolled, the enrollment of patients must be suspended immediately. Continuation of the study will then be subject to review by the Efficacy and Safety Evaluation Committee of the research group.

### Monitoring and quality assurance

The study entails a Data and Safety Monitoring Committee (DSMC) and committee members have a clear division of labor and cooperation. The supervision committee consists of a senior professor in the field of gastrointestinal surgery, data managers, data inspectors, medical ethics experts, and methodological teams. The DSMC will have unrestricted access to all research data and have the right to use monitors and auditors’ reports, as well as all other records related to quality assurance activities. The DSMC will periodically review the clinical efficacy and safety data collected in this study and evaluate the accumulated reports of SAEs. Concurrently, it can also implement emergency reviews and evaluations of safety-related issues. The study has a standard operation procedure (SOP) to ensure homogeneity of clinical data. Meanwhile, specialists are responsible for data collection and entry, data cleaning, and follow-up. After all the data are archived, researchers will submit data to the methodology team for statistical analysis. The DSMC is independent of the study sponsors, and there are no conflicts of interest.

### Sample size

The sample size was calculated using PASS 11.0 (NCSS Statistical and Data Analysis, USA) software and was estimated based on the results of our center’s previous research and published literature. According to our previous studies and domestic and foreign reports, the incidence of a mixed endpoint is approximately 20% for the “One-step” approach and approximately 45% for the “Step-up” approach [8]. Using a one-sided test, the α value was equal to 0.05, and the statistical power was 80%. The withdrawal rate was expected to be 20% during the study period. Thus, this study requires at least 128 patients, with 64 patients each group.

### Statistical analyses

The results of this trial for primary outcomes will be analyzed based on both the intention-to-treat protocol and per-protocol datasets. SAS 9.2 software (SAS Institute Inc.) was used to generate the randomization sequence. Statistical analyses will be performed with SPSS 21.0 (IBM Corp., Armonk, NY, USA) statistical software. The continuous variables with normal distributions will be described as mean ± standard deviation, and an independent sample *t-test* will be used for difference tests. The continuous variables with non-normal distributions will be described by the median (IQR), and the Mann–Whitney U test will be used for the difference tests. The Chi-square test or Fisher’s exact test will be used for categorical data. The Chi-square test or Fisher’s exact test will be used to assess adverse events. The Kaplan–Meier method will be used to calculate survival analysis. The log-rank test will be used to compare the complication rate between the two groups, and *P* values < 0.05 will be considered statistically significant.

### Interim analyses

Statistical analysis for the primary endpoint will be performed when the total number of enrolled patients reaches 64. The interim analysis will be carried out by an independent team of statisticians and the results will be reported to DSMC. The DSMC will have unrestricted access to all data and will discuss the results of the interim analysis and ultimately report to the Efficacy and Safety Evaluation Committee of the research group, who will determine whether the study can be continued or not. If the efficacy or safety of the “One-step” approach is lower than “Step-up” approach in the results of interim data, we will suspend the clinical trial.

### Patient and public involvement

The public was not involved in the study design or patient recruitment. The researchers played an important role in the study design, data collection, analysis, interpretation of data, writing of the report, and decision to submit the report for publication.

### Dissemination plans

We will publish the results in high-quality peer-reviewed journals at the end of the study.

### Trial status

Version 1.0 of the study protocol was approved in December 2020. The trial was registered in March 2021 on the Chinese Clinical Trial Registry website (http://www.chictr.org.cn/index.aspx). We established SOP and CRF for clinical research. Patient enrollment will begin in May 2021 and is anticipated to end in December 2023.

## Discussion

The treatment strategy of IPN is to perform adequate debridement and drainage under the premise of basic treatment, but there are some difficulties: (1) SAP patients are often in critical condition with organ dysfunction or serious complications, and have poor tolerance for surgical trauma. Therefore, timely and effective surgical treatment is often difficult, which delays the optimal treatment. (2) Even if laparotomy is performed, due to the great trauma, more serious complications such as bleeding, intestinal fistula, diffuse abdominal infection, or multiple organ dysfunction may worsen, with an average mortality rate of 30.8% [4, 13]. In recent years, with the rapid development of minimally invasive surgery, minimally invasive techniques have been gradually applied to the surgical treatment of IPN, and the therapeutic effect has significantly improved. The current minimally invasive treatment methods primarily include ultrasound-or CT-guided PCD, endoscopic pancreatic necrosis tissue debridement, laparoscopic abdominal cavity lavage, laparoscopic pancreatic or peripancreatic necrosis tissue debridement, and video-assisted retroperitoneal necrosis tissue debridement [5, 6, 14]. The PANTER study confirmed that the minimally invasive technique is significantly better than open surgery for the treatment of IPN, especially for critically ill patients, and has been recommended by various guidelines [5, 15, 16]. At present, minimally invasive surgery for IPN mostly adopts the “Step-up” treatment strategy, that is, PCD is performed first, and minimally invasive surgery and open surgery are performed sequentially for those whose infection symptoms cannot be controlled. In our clinical experience, we have observed some problems with the “Step-up” strategy. First, is PCD treatment necessary? PCD, as a minimally invasive treatment for rapidly controlling infection symptoms and improving the patient’s overall condition, has been adopted as the first choice of initial treatment for IPN in many clinical centers. However, some patients with IPN do not require PCD therapy because they are in good condition and can tolerate surgery after active systemic support. Secondly, the “Step-up” strategy may lead to longer treatment time and increased hospitalization costs. Previous research indicates that the treatment time of PCD is typically 1–3 weeks and the total course and cost of treatment for patients who experience treatment failure will significantly increase [17, 18]. Meanwhile, some patients lack a safe puncture path for PCD treatment. In addition, PCD treatment is associated with an increased risk of abuse. Since PCD is easy to master and results in few complications, it should be noted that there is no clinical tendency to choose the PCD procedure, which may lead to increased difficulty in subsequent surgical treatment. To overcome the above shortcomings, we put forward the concept of the “One-step” strategy in China, that is, minimally invasive video-assisted debridement is directly performed for patients with IPN patients who are in relatively good general condition without PCD procedure [8].

Moreover, the area of necrotic tissue and effusion of the IPN is closely related to the choice of minimally invasive operation methods and approaches and affects the therapeutic effect [19]. Previous studies on the distribution of AP fluid were based on the anatomical understanding of the pancreas or retroperitoneum, and it was believed that the distribution range of fluid was related to the severity of AP [20]. However, the above classification methods are complex and not practical, and there is currently a lack of direct correlation with the selection of minimally invasive surgery widely performed at present. Therefore, it is urgent to propose a clear diagnosis and treatment specification for the distribution characteristics of IPN and the selection of minimally invasive surgery, based on the results of prospective studies, so as to provide a reference for the treatment of IPN.

The limitation of this study is that although it is a randomized controlled study with high quality of evidence, participants are to be recruited only from among the Chinese population, and applications to other populations require further study. In addition, endoscopic transmural (transgastric or transduodenal) necrosectomy was not routinely used in this study. Therefore, it may not reflect the overall perspective of IPN treatment. However, we will publish the clinical data which related to endoscopic treatment in subsequent studies.

## Data Availability

We will transfer the CRFs to the EDC (https://edc.trialdata.cn/Member/Login), which will be stored in a hard disk and cloud system. The datasets used and/or analysed during the current study are available from the corresponding author on reasonable request.

## Abbreviations

IPN: Infected pancreatic necrosis
SAP: Severe acute pancreatitis
SIRS: Systemic inflammatory response syndrome
MODS: Multi-organ dysfunction
PCD: Percutaneous catheter drainage
CT: Computed tomography
ICU: Intensive care unit
WBC: White blood cell
IL-6: Interleukin-6
CRP: C-reactive protein
PCT: Procalcitonin
CRF: Case report form
EDC: Electronic data capture
CRA: Clinical research associate
CRC: Clinical research coordinator
SAE: Serious adverse events
DSMC: Data and safety monitoring committee
SOP: Standard operation procedure
POD: Postoperative day
